# Late presenters: what can we do?

**DOI:** 10.7448/IAS.15.6.18072

**Published:** 2012-11-11

**Authors:** C Mussini

**Affiliations:** University of Modena, Modena, Italy

## Abstract

Late presentation represents a major problem for patients with HIV infection. Actually, it should be made a distinction between late testers and late presenters since the strategies to reduce the percentage of these two groups of subjects could be different. Indeed, the first population is represented by individuals unaware of their serological status, while in the second case the problem is related to engagement and retention in care. Concerning the first population, it has been shown that most of the patients had been seen by their family doctor or admitted to hospital during the year before HIV diagnosis. Indeed, this is a relevant problem, and new strategies to increase the level of suspicion of HIV infection among doctors who are non-HIV specialists are needed, as testing in presence of indicator diseases, should be applied. Concerning the population of late presenters, American data showed a percentage of engagement in care ranging between 50 and 59%. These low percentages could be due to the American Health System, while in a public health system setting, the percentage of patients not engaged in care or lost to follow-up could be lower, even if still relevant. Another important factor that should be considered in both populations is stigma. Indeed, many patients that present late, either late testers or late presenters, are immigrants and have important cultural barriers to disclose their HIV serostatus to family members and friends. Obviously, all subjects unaware or refusing their HIV infection could potentially infect their partners. In conclusion, all efforts should be made to reduce the phenomenon of late presentation since these two populations represent an epidemiological problem not only for the prognosis of the single patient but also for the treatment as prevention strategy.

